# Notch Is a Critical Component of the Mouse Somitogenesis Oscillator and Is Essential for the Formation of the Somites

**DOI:** 10.1371/journal.pgen.1000662

**Published:** 2009-09-25

**Authors:** Zoltan Ferjentsik, Shinichi Hayashi, J. Kim Dale, Yasumasa Bessho, An Herreman, Bart De Strooper, Gonzalo del Monte, Jose Luis de la Pompa, Miguel Maroto

**Affiliations:** 1 Division of Cell and Developmental Biology, College of Life Sciences, University of Dundee, Dundee, Scotland, United Kingdom; 2 Graduate School of Biological Sciences, Nara Institute of Science and Technology, Ikoma, Nara, Japan; 3 Department of Molecular and Developmental Genetics, Vlaams Instituut voor Biotechnologie, Leuven, Belgium; 4 Center for Human Genetics, KULeuven, Leuven, Belgium; 5 Cardiovascular Developmental Biology Department, Centro Nacional de Investigaciones Cardiovasculares, Madrid, Spain; University of Maine, United States of America

## Abstract

Segmentation of the vertebrate body axis is initiated through somitogenesis, whereby epithelial somites bud off in pairs periodically from the rostral end of the unsegmented presomitic mesoderm (PSM). The periodicity of somitogenesis is governed by a molecular oscillator that drives periodic waves of clock gene expression caudo-rostrally through the PSM with a periodicity that matches somite formation. To date the clock genes comprise components of the Notch, Wnt, and FGF pathways. The literature contains controversial reports as to the absolute role(s) of Notch signalling during the process of somite formation. Recent data in the zebrafish have suggested that the only role of Notch signalling is to synchronise clock gene oscillations across the PSM and that somite formation can continue in the absence of Notch activity. However, it is not clear in the mouse if an FGF/Wnt-based oscillator is sufficient to generate segmented structures, such as the somites, in the absence of all Notch activity. We have investigated the requirement for Notch signalling in the mouse somitogenesis clock by analysing embryos carrying a mutation in different components of the Notch pathway, such as *Lunatic fringe* (*Lfng*), *Hes7*, *Rbpj*, and *presenilin1*/*presenilin2* (*Psen1*/*Psen2*), and by pharmacological blocking of the Notch pathway. In contrast to the fish studies, we show that mouse embryos lacking all Notch activity do not show oscillatory activity, as evidenced by the absence of waves of clock gene expression across the PSM, and they do not develop somites. We propose that, at least in the mouse embryo, Notch activity is absolutely essential for the formation of a segmented body axis.

## Author Summary

Vertebrate animals generate their segmented body plan during embryogenesis. These embryonic segments, or somites, form one after another from tissue at the tail end of the embryo in a highly regulated process controlled by a molecular oscillator. This oscillator drives the expression of a group of genes in this tissue and determines the periodicity of somite formation. To date the genes regulated by this molecular clock comprise components of the Notch, Wnt, and FGF pathways. Recent data in the zebrafish embryo have suggested that the only role of Notch signalling in this process is to synchronise gene oscillations between neighbouring cells and that somite formation can continue in the absence of Notch activity. However, we show that mouse embryos lacking all Notch activity do not show oscillatory activity and do not develop somites. We propose that, at least in the mouse embryo, Notch activity is absolutely essential for building a segmented body axis.

## Introduction

Segmentation is a key feature of the body plan of all vertebrates, including humans, that initiates very early in embryonic development. The first sign of metamerism or segmentation is seen when vertebrate embryos develop somites, the precursors of several segmented organs such as the axial skeleton, body skeletal muscles and part of the dermis. Somites are formed in a highly regulated process called somitogenesis from the unsegmented presomitic mesoderm (PSM) [1]–[3]. During the formation of somites the most mature PSM cells located at the rostral end of the PSM bud off as an epithelial sphere of cells to form the somite. Somite formation occurs simultaneously with the recruitment of newly generated mesenchymal cells from the primitive streak/tail bud into the caudal region of the PSM [2]–[4].

Critical molecular and embryological experimental data obtained in the last ten years has shown that somitogenesis is governed by a molecular oscillator [5] that drives cyclic expression of genes in the PSM and which is coupled to the formation of the somites [2], [3], [6]–[8]. Expression of these cyclic genes is coordinated such that a wave of expression travels caudo-rostrally throughout the PSM during the formation of one somite. All cyclic genes identified to date encode either (a) components or modulators of the Notch pathway (b) components of the Wnt pathway or (c) components of the FGF pathway [2], [3], [6]–[8].

There are discrepancies in the literature regarding the role(s) of Notch signalling during the process of somite formation. At least in the zebrafish embryo it seems clear that Notch signalling has a predominant function in the synchronization of clock gene oscillations, where inhibition of Notch is not sufficient to interrupt the generation of a segmented body plan [7]–[11]. This view of Notch as a clock synchronizer has also been proposed to operate during mouse somitogenesis [12],[13]. On the other hand, data generated in chick, mouse and zebrafish is consistent with Notch being an important component of the molecular oscillator in different vertebrate species. Thus, ectopic expression of Lfng in the chick PSM or morpholino treatment against *her* genes in zebrafish embryos interferes with cyclic gene expression and leads to the generation of irregular somites, similar to the phenotype observed in different mouse and zebrafish transgenic lines carrying a mutation in various components of the Notch pathway [2],[3],[8]. Finally, a third possibility is that Notch signalling may have dual functions as both a clock generator as well as a clock synchronizer [14],[15]. In this report we re-examine the implication of Notch signalling in the mechanism of the mouse somitogenesis oscillator and in murine somite formation by analysing embryos carrying a mutation in different components of the Notch pathway, such as *Lunatic fringe* (*Lfng*), *Hes7*, *Rbpj* and *presenilin1*/*presenilin2* (*Psen1*/*Psen2*), and by pharmacological blocking of the Notch pathway. Our results show that, at least in the mouse embryo, Notch activity, be it cyclic or non-cyclic, is critically required both for the generation of periodic transcription of cyclic genes by the somitogenesis oscillator and for the formation of the somites.

## Results

### Mouse embryos can generate somites in the absence of Lfng or Hes7

**Figure 1:**
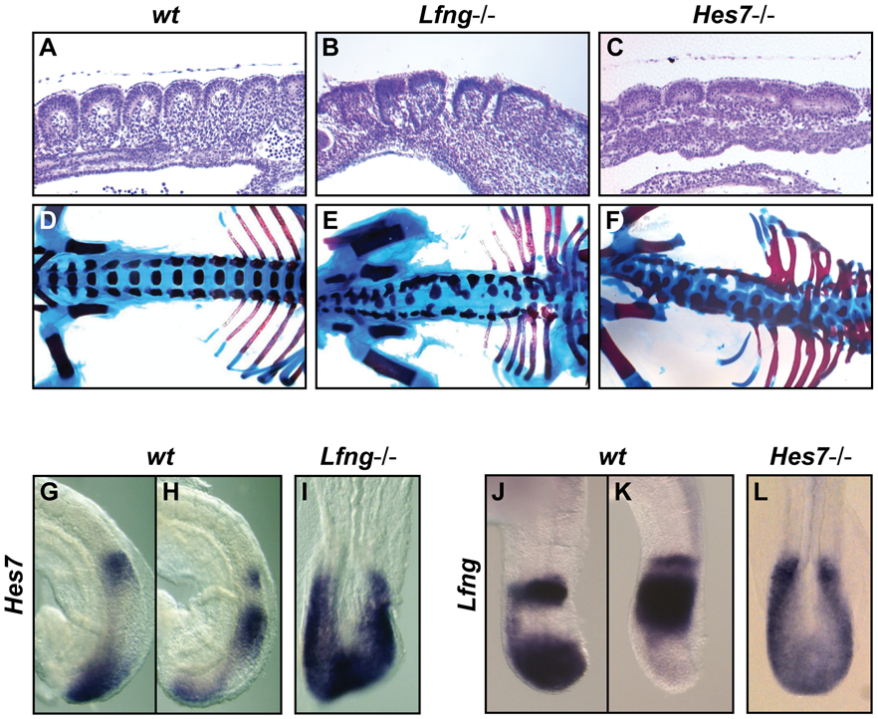
*Lfng*−/− and *Hes7*−/− embryos can develop a segmented body axis. (A–C) Parasagittal sections of the trunk region of an E9.5–10.5 (A) wild type, (B) *Lfng*−/− and (C) *Hes7*−/− embryo showing the irregular somites that have formed in the mutant embryos. (D–F) Alcian Blue/alizarin red stained E18.5 (D) wild type, (E) *Lfng*−/− and (F) *Hes7*−/− embryos showing the skeletal abnormalities along the spinal column in the mutant embryos. (G,H,J,K) Lateral and (I,L) dorsal views of E9.5–10.5 embryos analysed by *in situ* hybridisation using (G–I) *Hes7* or (J–L) *Lfng* probes. (G,H) Two wild type embryos displaying different pattern of *Hes7* expression and (I) one *Lfng*−/− embryo showing *Hes7* expression throughout the PSM. (J,K) Two wild type embryos displaying different pattern of *Lfng* expression and (L) one *Hes7*−/− embryo showing *Lfng* expression throughout the PSM.

To further clarify the role of Notch signalling during mouse somitogenesis we decided to analyse in detail the phenotype of two mouse knockout lines, namely *Hes7*−/− [16] and *Lfng*−/− [17],[18], two components of the Notch pathway. Hes7 is a downstream target of Notch and encodes a repressor of transcription previously shown to be a negative component of the machinery of the somitogenesis oscillator [19],[20]. Lfng is also a downstream target of Notch that encodes a glycosyltransferase that modulates the potential of the Notch receptor protein to interact with its ligands Delta and Serrate/Jagged [21]. We first analysed the organization of the somites in E9.5–10.5 homozygous null embryos of both mutant lines, *Lfng*−/− (n = 5) and *Hes7*−/− (n = 6), and observed that they are irregular, their size is not uniform, they are occasionally fused (Figure 1A–1C) and sometimes they display left-right asymmetry (data not shown). We observed these disordered somites in the mutant embryos over a variety of developmental stages, E8.5–10.5 (data not shown), as previously reported [16],[20]. To visualise the long term impact of the absence of these two Notch-related components of the oscillator in the formation of the metameric body plan we performed alcian blue/alizarin red staining, which stains all bones and cartilage, using E18.5 mouse embryos. As expected, *Lfng*−/− (n = 4) and *Hes7*−/− (n = 4) embryos displayed skeletal abnormalities along the spinal column (Figure 1D–1F; [16]–[18]). Thus, in the absence of Lfng or Hes7 the process of somitogenesis is not properly regulated. However, of more relevance for this particular analysis is the fact that these embryos are capable of producing somites and vertebrae at all, which indicates the potential existence of periodic activity produced by the somitogenesis oscillator machinery.

To address the nature of this oscillator we analysed the expression of a number of oscillatory genes. Initially we analysed the expression of Notch-regulated cyclic genes. The expression of *Hes7* was upregulated in the entire PSM of *Lfng−/−* embryos (n = 10, Figure 1I). Similarly, we analysed *Hes7*−/− embryos and observed that they also displayed upregulated expression of *Lfng* (n = 20, Figure 1L; [16]). Thus, the results show that in the absence of important negative regulatory components, such as Hes7 and Lfng, Notch activity appears upregulated even if the mutant embryos are still able to generate segmented structures.

### Notch activity is still dynamic in the PSM of *Lfng*−/− embryos

**Figure 2:**
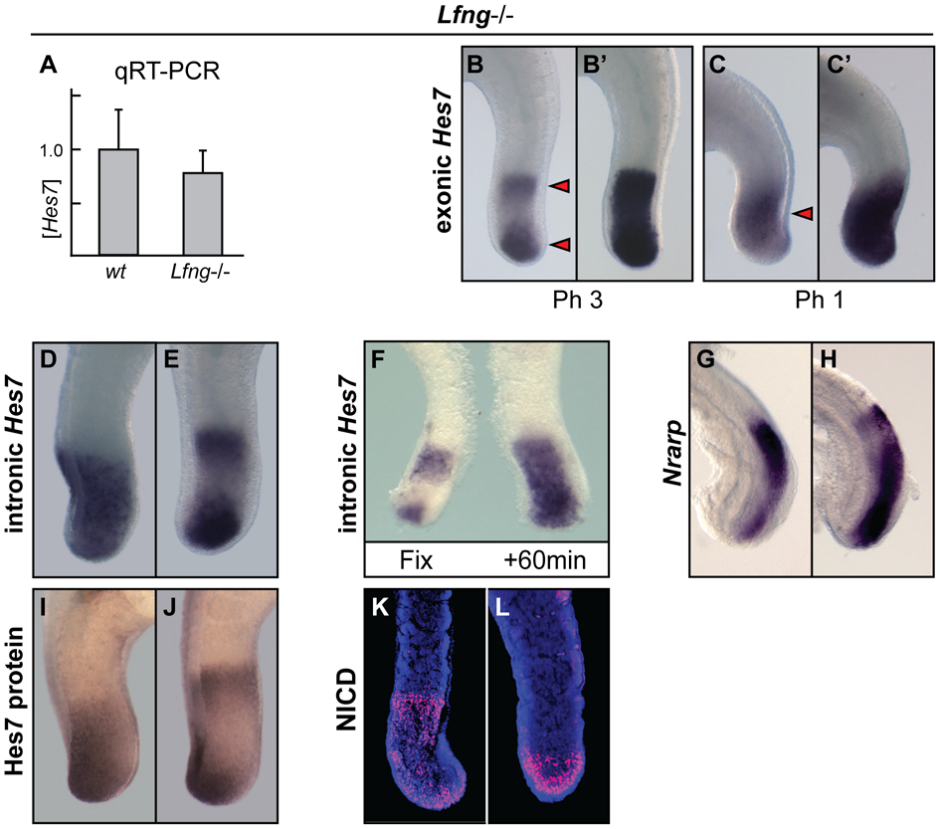
Notch-based cyclic gene expression is still dynamic in the PSM of *Lfng*−/− embryos. (A) Bar chart showing that the relative amount of *Hes7* mRNA in the PSM of wild type and *Lfng*−/− embryos is not statistically different as judged by qRTPCR (P = 0.130). Error bars represent standard deviation. (B–L) Lateral views of the caudal region of E9.5–10.5 *Lfng*−/− embryos analysed by *in situ* hybridisation or immunocytochemistry using (B,B',C,C') *Hes7*, (D–F) intronic *Hes7*, (G,H) and *Nrarp* probes, (I,J) anti-Hes7 or (K,L) anti-NICD antibodies. (F) Fix and culture assay using E10.5 caudal explants of *Lfng*−/− embryo confirms oscillatory *Hes7* expression. Arrowheads demarcate distinct domains of expression in the PSM.

**Figure 3:**
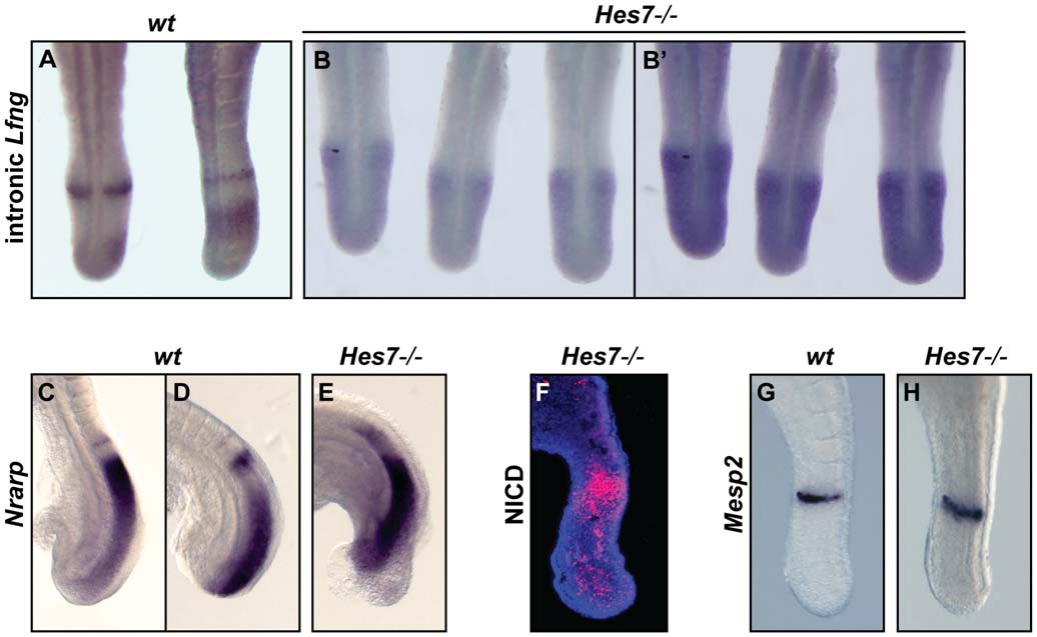
Notch activity is present but Notch-based cyclic gene expression is not dynamic in the PSM of *Hes7*−/− embryos. (A,B,B') Dorsal and (C–H) lateral views of E9.5–10.5 (A,C,D,G) wild type or (B,B',E,F,H) *Hes7*−/− embryos analysed by *in situ* hybridisation or immunocytochemistry using (A,B,B') an intronic *Lfng* probe, (C–E) an *Nrarp* probe, (F) an anti-NICD antibody and (G,H) a *Mesp2* probe. (B,B') *Lfng* and (F) NICD do not show different patterns of expression in the PSM of *Hes7*−/− embryos.

In principle, the observed upregulation of Notch downstream targets in the *Lfng*−/−embryos could culminate in an accumulation of the mRNA for these genes along the PSM. To test this, we decided to measure the amount of *Hes7* mRNA present in the rostral half of the PSM of wild type or *Lfng−/−* embryos. To that end we isolated the total RNA from pooled rostral half PSM samples of several wild type and mutant embryos of unknown cyclic phases and then performed quantitative RT-PCR. We observed that the relative expression level of *Hes7* revealed no statistically significant difference between wild type (n = 12) and *Lfng−/−* (n = 10) PSM samples (Figure 2A; t-test, df = 20, P = 0.130). One possible explanation for this lack of accumulation of *Hes7* mRNA in the *Lfng*−/− embryos might be that the transcripts are in fact produced and degraded as in the wild type embryo, albeit not quite as efficiently. We decided to re-examine *Hes7* mRNA expression in these *Lfng*−/− embryos in more detail. When we re-analysed *Lfng−/−* embryos with the *Hes7* probe carefully monitoring the intensity of the revelation step we observed different patterns or phases of expression (n = 13, Figure 2B and 2C; [22]). Longer staining of the same mutant embryos led to the general upregulation of *Hes7* described above (Figure 2B' and 2C', Figure 1I). Under similar conditions of longer staining this general upregulation is not observed using wild type embryos (n = 25, data not shown). To further corroborate these data we analysed *Lfng*−/− embryos using a *Hes7* intronic probe in order to detect nascent pre-spliced mRNA and thereby to show the location of active transcription [23]. The *Lfng*−/− embryos (n = 6) presented patterns of *Hes7* expression similar to those observed in wild type embryos (n = 6, Figure 2D and 2E, data not shown). To confirm that these different patterns corresponded indeed to a dynamic activity we performed a half embryo analysis, in which the tail of an embryo is split longitudinally in two halves, then one half is immediately fixed and the other is cultured for 60 minutes before fixation [24],[25]. *In situ* hybridisation with an intronic *Hes7* probe on samples prepared using this type of analysis showed that the two halves displayed different patterns of expression (n = 5, Figure 2F), which clearly indicates that in the absence of Lfng activity the expression of the Notch-related cyclic gene *Hes7* is still dynamic. Similarly, when we analysed the expression of a second Notch-related cyclic gene, *Nrarp*, we also found different patterns of expression similar to those observed in wild type embryos (n = 8 and n = 12 respectively, Figure 2G and 2H, Figure 3C and 3D; [26]). In addition, we analysed the expression of Hes7 protein in tails of E10.5 *Lfng*−/− embryos (n = 8) using a specific anti-Hes7 antibody [19] and found that Hes7 protein also displayed different phases of expression, consistent with it being cyclic, although the boundaries of expression were not as sharp as in the wild type (n = 8 and n = 10 respectively, Figure 2I and 2J; data not shown). Finally, we tested the expression of the cleaved intracellular portion of Notch (NICD), which is the active fragment of Notch and is generated when Notch receptor, after its interaction with the ligand, is processed by the γ-secretase complex. Once NICD is produced it translocates to the nucleus where it binds to RBPj and the complex becomes a transcriptional activator of downstream targets [21]. NICD has previously been reported to display a dynamic expression profile in the PSM of wild type mice embryos [27],[28]. Using an antibody specific for NICD we stained tail sections of E10.5 *Lfng*−/− embryos and observed different patterns of NICD expression (n = 9, Figure 2K and 2L), consistent with Notch activity still being dynamic. These results clearly indicate that in the absence of the glycosyltransferase Lfng it is possible to detect dynamic NICD and dynamic expression of Notch-related cyclic genes, although not with sharp boundaries of expression, which suggests that Lfng could be an important but not a critical component of the mouse oscillator.

### Oscillations of Notch cyclic genes are absent in the PSM of *Hes7*−/− embryos but not those of Wnt and FGF cyclic genes

In order to examine more closely whether the *Hes7−/−* embryos also retained some cyclic Notch activity, we first analysed these embryos by *in situ* hybridisation with an intronic probe against the Notch-regulated cyclic gene *Lfng*. Strikingly, we found a different situation in the *Hes7−/−* embryos than we had observed in the *Lfng*−/− embryos and we were not able to detect the existence of different phases of *Lfng* expression. During the course of a controlled staining all *Hes7−/−* embryos (n = 10) displayed the same profile of a broad rostro-caudal gradient of expression (Figure 3B). Longer staining of the same mutant embryos led to the general upregulation of *Lfng* described above (Figure 3B', Figure 1I). Similarly, the expression pattern of the Notch-regulated cyclic gene *Nrarp* was identical in all *Hes7*−/− embryos (n = 7, Figure 3E). These non-dynamic patterns are consistent with the fact that in these *Hes7*−/− embryos we did not see different patterns of NICD expression either, rather it was detected in a rostro-caudal gradient of expression across the PSM (n = 6, Figure 3F). Expression of the non-cyclic Notch target gene *Mesp2* [29],[30] was retained in the rostral region of the PSM of *Hes7*−/− embryos, although the expression domain was not as sharp as in wild type embryos (Figure 3G and 3H; [16]). It has been reported that *Mesp2* expression is severely compromised in *Dll1*−/− and *Rbpj*−/− mutant embryos [31]. Thus, the *Mesp2* band of expression observed in the *Hes7*−/− embryos is probably due to the presence of non-dynamic Notch signalling activity in the PSM. In summary, there is a non-dynamic expression of Notch-based cyclic genes in the PSM of *Hes7*−/− embryos, which mirrors the non-dynamic Notch activity in this tissue.

**Figure 4:**
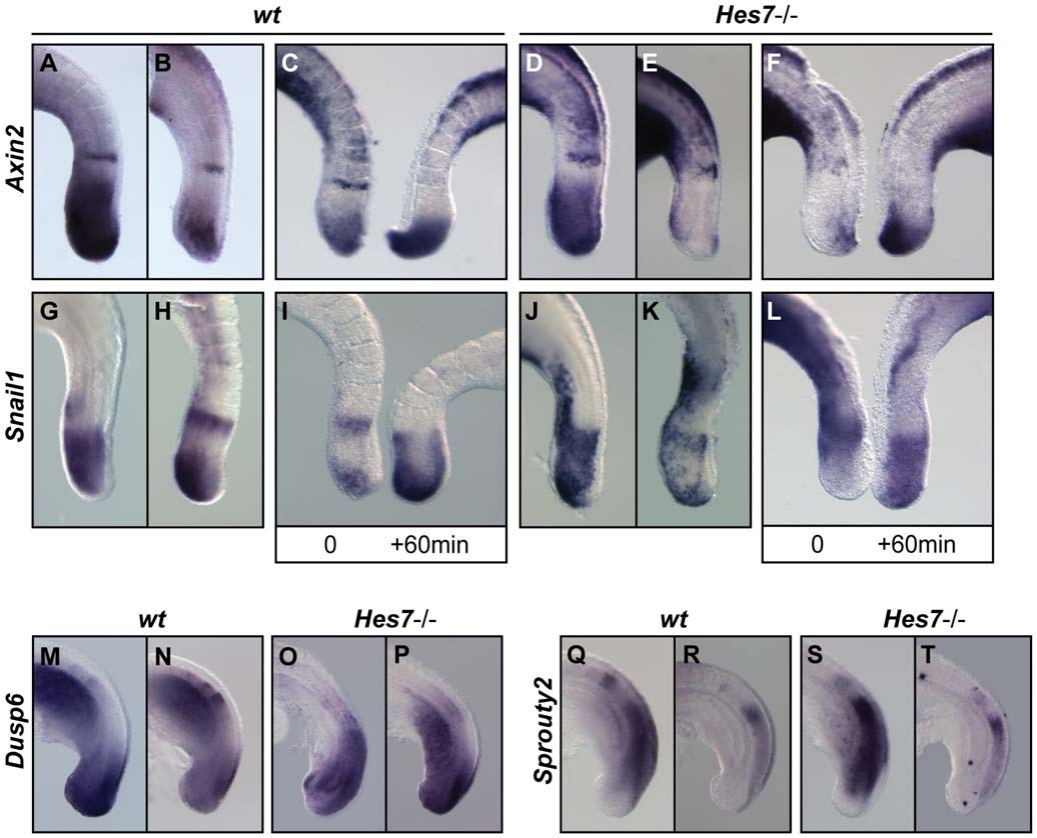
Wnt/FGF-based cyclic gene expression is dynamic in the PSM of *Hes7*−/− embryos. Lateral views of E9.5–10.5 (A–C,G–I,M,N,Q,R) wild type or (D–F,J–L,O,P,S,T) *Hes7*−/− embryos analysed by *in situ* hybridisation using (A–F) *Axin2*, (G–L) *Snail1*, (M–P) *Dusp6* and (Q–T) *Sprouty2* probes. (F,L) Fix and culture assay using E10.5 caudal explants of *Hes7*−/− embryos confirms oscillatory *Axin2* and *Snail* expression.

Since in the *Hes7−/−* embryos, unlike the *Lfng−/−* embryos, there does not appear to be cyclic activity of Notch-regulated cyclic genes we investigated whether any of the FGF or Wnt regulated cyclic genes were oscillating in the PSM of these *Hes7−/−* embryos. Based on the patterns of expression observed in *Dll1*−/− embryos it has been proposed that the expression of *Axin2* is independent of Notch activity [32]. We first examined the expression of the Wnt-related cyclic gene *Axin2* in the PSM of *Hes7*−/− embryos (n = 30) and observed different patterns of expression (Figure 4D and 4E; [20]). In addition, a fix and culture analysis clearly indicated that this *Axin2* expression is dynamic in the *Hes7−/−* mutant background (n = 21, Figure 4F). We also analysed the expression of the FGF/Wnt-regulated gene *Snail1* by *in situ* hybridisation (n = 10) and fix and culture (n = 5) and observed that it is also dynamic (Figure 4J–4L; [33]). Our results indicate that the *Hes7*−/− embryo makes irregular somites in the absence of cyclic Notch but in the presence of cyclic Wnt activity. The third pathway described to be a critical component of the murine somitogenesis oscillator is the FGF pathway. FGF is reported to be responsible for the initiation of *Hes7* expression in the caudal PSM [34]–[36] and some components of the FGF pathway, such as *Dusp4/6* and *Sprouty2/4*, have been shown to display dynamic expression in the PSM [34],[37],[38]. We tested the expression of *Dusp6* (n = 30) and *Sprouty2* (n = 32) in *Hes7−/−* embryos and observed different patterns of expression, consistent with dynamic FGF activity in the PSM of these embryos (Figure 4O, 4P, 4S, and 4T). In summary, we conclude that embryos lacking *Hes7* retain dynamic activity of the Wnt-regulated genes and FGF-regulated genes of the somitogenesis oscillator, which is likely to underlie the generation of periodicity and the formation of irregular somites in these embryos.

### Somitogenesis is lost in the *Psen1/Psen2* double mutant embryos

**Figure 5:**
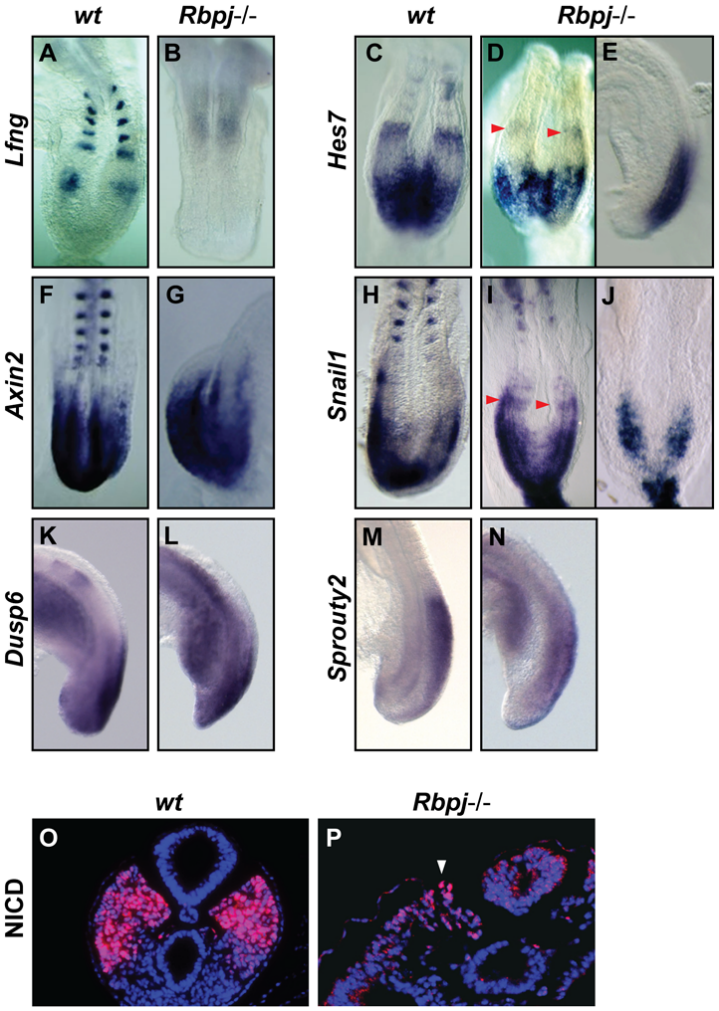
Notch-based cyclic gene *Hes7* displays different patterns of expression in the PSM of the *Rbpj*−/− embryos. (A–J) Dorsal and (K–N) lateral views of caudal region of E8.5–9.5 (A,C,F,H,K,M) wild type or (B,D,E,G,I,J,L,N) *Rbpj*−/− embryos analysed by *in situ* hybridisation using (A,B) *Lfng* and *Uncx4.1*, (C–E) *Hes7*, (F,G) *Axin2* and *Uncx4.1*, (H–J) *Snail1* and *Uncx4.1*, (K,L) *Dusp6* and (M,N) *Sprouty2* probes. (O,P) Immunostaining prepared using the anti-NICD antibody on cross-sections of E8.5 (O) wild type and (P) *Rbpj*−/− embryos showing expression in the PSM. Red arrowheads demarcate a distinct, discrete rostral domain of expression of *Hes7* in the PSM of the mutant embryo. White arrowhead indicates the weak NICD expression detected in the PSM of the mutant embryo.

Our data indicate that in *Lfng*−/− embryos the cyclic gene oscillations comprise elements of the Notch pathway (Figure 2). This is in contrast to the situation in the *Hes7*−/− embryos because the oscillatory mechanism appears to be based only on Wnt and FGF-dependent genes. It is not clear, however, if this Wnt/FGF-based oscillator is sufficient to generate segmented structures, such as the somites, in the complete absence of all Notch activity, since in these *Hes7*−/− embryos there remains clear evidence of non-dynamic Notch activity, which could be a critical requirement for the proper function of an Wnt/FGF-based somitogenesis oscillator and/or the formation of the somites. To more definitively test the relevance of Notch activity during the process of somitogenesis we decided to re-analyse homozygous null embryos from two other mutant lines widely accepted to develop in the complete absence of Notch activity, *Rbpj*−/− and *Psen1*−/−;*Psen2*−/− [39]–[41]. We first evaluated the situation in *Rbpj*−/− embryos. RBPj is the transcriptional repressor to which NICD binds in the nucleus in order to activate expression of downstream target genes [21],[42],[43]. *Rbpj*−/− embryos die at approximately E9.5 after forming a variable number (zero to five) of disorganized and irregular somite-like structures [39]. As expected, we observed that at stage E8.5–9.0 the expression of the Notch-regulated cyclic gene *Lfng* (n = 6) was lost in the PSM (Figure 5B). Barrantes and colleagues have previously reported that in a few cases they were able to detect a single faint stripe of *Lfng* in the rostral PSM [31]. Surprisingly, however, we found that the Notch-related cyclic gene *Hes7* was still present along the PSM of these *Rbpj*−/− embryos (n = 5, Figure 5D and 5E; [34]). In fact, *Hes7* expression can be detected with different patterns of expression in a broad caudal domain and in restricted bands in the rostral region, similar to the expression observed in wild type embryos (Figure 5C), suggesting that its dynamic character may still be functional in these mutant embryos. Similarly we also found that the Wnt/FGF-based cyclic gene *Snail1* displayed different patterns of expression, including both a caudal domain and a rostral band of expression (n = 14, Figure 5I and 5J; [33]). The Wnt-related cyclic gene *Axin2* (n = 4) and the FGF-related cyclic genes *Dusp6* (n = 4) and *Sprouty2* (n = 5) were also found expressed along the PSM with patterns of expression similar to those found in wild type embryos (Figure 5G, 5L, and 5N). Of critical importance to this study is the fact that in *Rbpj*−/− embryos two Notch-dependent cyclic genes, *Lfng* and *Hes7*, respond differently to the absence of RBPj activity and at least *Hes7* displays patterns of expression in the PSM similar to those observed in the wild type, which may be dynamic. These data raise the question of whether these mutant embryos do in fact develop in the complete absence of Notch activity or whether there remains some residual RBPj-independent Notch activity similar to what has been described in *Drosophila* [44],[45]. Feller and colleagues have shown that the expression of these two Notch targets, *Lfng* and *Hes7*, is also differentially affected following perturbation of Notch activity in the PSM of embryos expressing constitutive-activate Notch (T-NICD) [13]. Thus, it would appear that they are not equally sensitive to the levels of NICD as an input to their expression. It is formally possible that the absence of RBPj results in a severe decrease of Notch signalling leading to a loss of *Lfng* but that a certain level of RBPj-independent NICD activity remains, which could act to maintain the expression of *Hes7*. To further investigate this possibility we decided to explore if NICD is expressed in the PSM of the *Rbpj*−/− embryos. Our immunostaining on sections clearly indicate that indeed it is possible to detect weak NICD expression in the PSM of these mutant embryos (Figure 5P; [46]). From these two results, the existence of different patterns of expression of *Hes7* in the PSM and the expression of NICD, we conclude that the *Rbpj*−/− mutant line is not appropriate to definitively test the significance of a complete lack of Notch activity during the process of somitogenesis.

**Figure 6:**
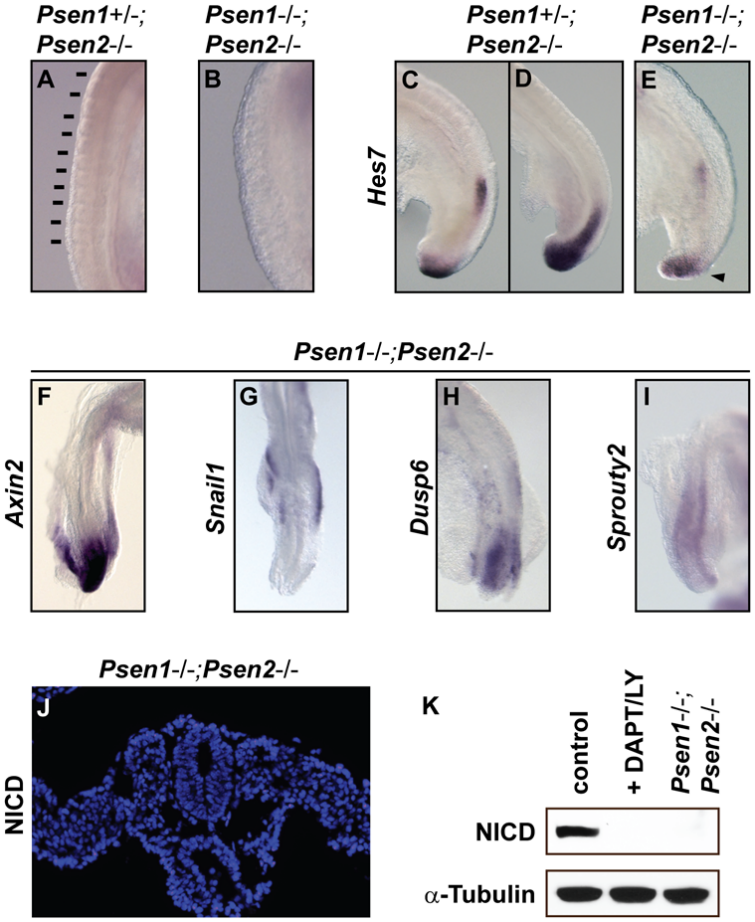
Cyclic gene expression is lost or restricted to the caudal PSM in *Psen1*/*Psen2* double mutant embryos. (A–E) Lateral and (F–I) dorsal views of caudal regions of E9.5 (A,C,D) *Psen1+/−;Psen2−/−* or (B,E,F–I) *Psen1−/−;Psen2−/−* double mutant embryos analysed by *in situ* hybridisation using (C–E) *Hes7*, (F) *Axin2*, (G) *Snail1*, (H) *Dusp6* and (I) *Sprouty2* probes. (J) Immunostaining using the anti-NICD antibody on cross-sections of an E9.5 *Psen1*−/−;*Psen2*−/− embryo showing no expression in the PSM. (K) western blot analysis with control embryonic extract, embryonic extract after treatment with DAPT/LY411575 and embryonic fibroblast prepared from *Psen1*−/−;*Psen2*−/− embryos analysed with and anti-α-tubulin and anti-NICD antibodies. Black arrowhead demarcates a distinct, discrete caudal domain of expression of *Hes7* in the PSM.

We next examined embryos from the double knockout line *Psen1*/*Psen2*, which generate mutant embryos lacking all presenilin activity [40],[41]. Presenilin is the catalytic component of the γ-secretase complex responsible for the cleavage of Notch receptor and the generation of NICD. In principle, this mutant mouse line should lack all Notch activity. *Psen1*+/−;*Psen2*−/− embryos retaining one allele of presenilin1 did not display a somitic phenotype (n = 20, Figure 6A) and showed normal patterns of *Hes7* expression along the PSM (Figure 6C and 6D). In contrast, *Psen1*−/−;*Psen2*−/− embryos lacking the two presenilins failed to form any somites (n = 22, Figure 6B; [40]) and *Hes7* expression was absent in the medial and rostral PSM, consistent with this expression domain being entirely Notch dependent. Nevertheless, it was possible to detect weak *Hes7* expression in the tail bud region (n = 4, Figure 6E), which may be indicative of FGF-induced activation in this domain since FGF is reported to be responsible for the initiation of *Hes7* expression in the caudal PSM [34],[35]. Consistent with the idea that these double mutant embryos develop in the complete absence of Notch activity is the fact that NICD was not detected by immunostaining on sections prepared from these double mutant embryos (Figure 6J). To confirm this negative result we also performed western blot analysis using protein samples prepared with embryonic fibroblasts from the *Psen1*−/−;*Psen2*−/− embryos [47] and observed that NICD is not produced (Figure 6K; [48]). These data indicate that, in contrast to our observations in the *Rbpj*−/− embryos, the *Psen1*−/−;*Psen2*−/− embryos develop in the complete absence of Notch activity, as judged by the absence of NICD production and the absence of cyclic expression of Notch-dependent *Hes7*. Interestingly, we also found that in these double mutant embryos the expression of *Axin2* (n = 4), *Snail1* (n = 4), *Dusp6* (n = 4) and *Sprouty2* (n = 4) is lost along the PSM and when they are detected the remaining staining is restricted to the neural tube or caudal tail bud (Figure 6F–6I). Together these results indicate that the Notch activity appears to be critical both to maintain any form of dynamic clock gene expression in the PSM and to generate somites.

### Pharmacological elimination of Notch activity in the mouse PSM abolishes somitogenesis and all cyclic gene expression

**Figure 7:**
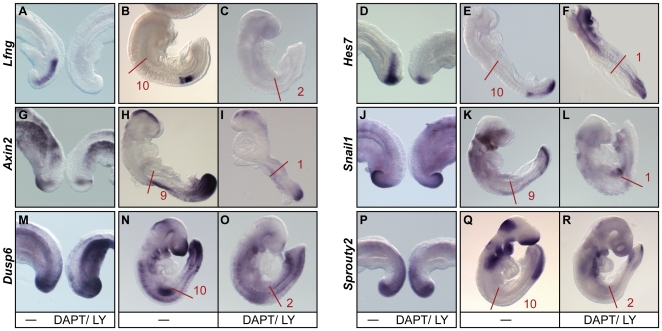
Cyclic gene expression and somite formation are lost after Notch-blocking drug treatment. (A,D,G,J,M,P) Half embryo explants from E9.5–10.5 wild type embryos cultured *in vitro* in the absence (left half) or presence (right half) of 100 µM of DAPT or 100 nM LY411575 for 3 hours and then analysed by *in situ* hybridisation for the expression of (A) *Lfng*, (D) *Hes7*, (G) *Axin2*, (J) *Snail1*, (M) *Dusp6* and (P) *Sprouty2*. (B,C,E,F,H,I,K,L,N,O,Q,R) Lateral views of E8.0–8.5 wild type embryos cultured in a roller culture system in the absence (B,E,H,K,N,Q) or presence (C,F,I,L,O,R) of 100 µM of DAPT or 100 nM LY411575 for 18–20 hours and then analysed by *in situ* hybridisation for the expression of (B,C) *Lfng*, (E,F) *Hes7*, (H,I) *Axin2*, (K,L) *Snail1*, (N,O) *Dusp6* and (Q,R) *Sprouty2*, showing that cyclic gene expression and somite formation are interrupted after drug treatment. Red bars demarcate the limit of the somites already formed at start of drug treatment and number indicates the somites formed during culture in the absence or presence of the drug.

**Figure 8:**
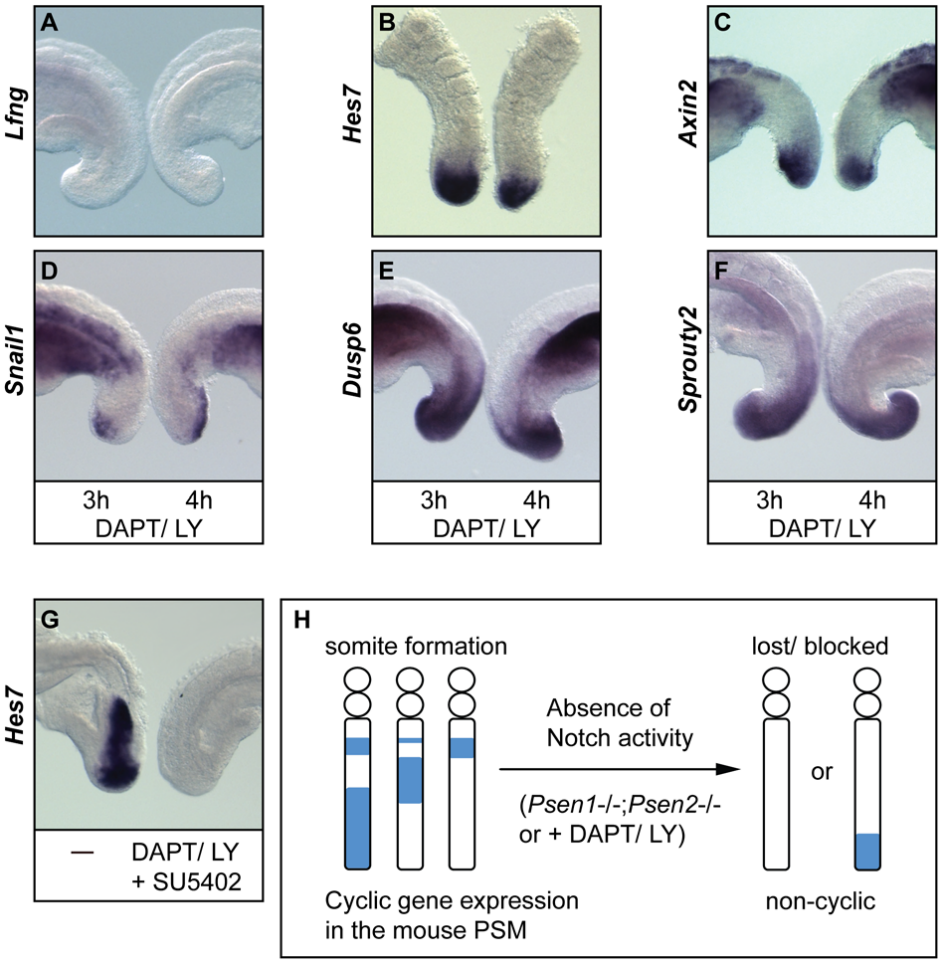
Cyclic gene expression in the tail bud is lost or stops being dynamic after treatment with Notch-blocking drugs. (A–F) Half embryo explants from E9.5–10.5 wild type embryos cultured *in vitro* in the presence of 100 µM of DAPT or 100 nM LY411575 for 3 hours (left) or 4 hours (right) and then analysed by *in situ* hybridisation for the expression of (A) *Lfng*, (B) *Hes7*, (C) *Axin2*, (D) *Snail1*, (E) *Dusp6* and (F) *Sprouty2*, showing that after drug treatment the cyclic gene expression is lost in the medial and rostral PSM, and that remaining expression still present in the tail bud is not dynamic. (G) Half embryo explants from E9.5 wild type embryos cultured *in vitro* in the absence (left half) or presence (right half) of 100 µM of DAPT plus 50 µM SU5402, a FGF signalling inhibitor, and then analysed by *in situ* hybridisation for the expression of *Hes7*. (H) Schematic representation of the data from the *Psen1*−/−;*Psen2*−/− embryos and the pharmacological treatment with Notch-blocking drugs showing that the oscillations of all cyclic genes along the PSM detected in wild type or untreated embryos are lost in the absence of Notch signalling. The expression of cyclic genes is completely lost or is severely down regulated and restricted to the caudal region of the PSM where it is non dynamic. In addition, in *Psen1*−/−;*Psen2*−/− embryos or in wild type embryos treated with Notch-blocking drugs the formation of somites is lost.

We decided to confirm these observations by blocking all Notch activity in the PSM using a pharmacological treatment and then analysed the effect of this treatment on the expression of oscillatory genes and somite formation, similar to the approach used in recent studies focussing on the role of Notch during somitogenesis in the zebrafish embryo [6],[10],[11],[49]. We first performed the treatment using the half embryo assay in the presence or absence of either 100 µM DAPT or 100 nM LY411575, two reagents that block Notch by inhibiting the γ-secretase cleavage of the Notch receptor [50]–[54]. We confirmed by western blot that after a 3 hour exposure to the Notch-blocking drugs the expression of NICD was abrogated (Figure 6K), as previously reported [48],[55]. Furthermore the expression of *Lfng* and *Nrarp* was completely abolished from the PSM, as expected (n = 8, Figure 7A; [24],[25]; Wright *et al.*, submitted). In addition, the drug-treated samples showed no expression of *Hes7* in the rostral PSM, consistent with this expression domain being entirely Notch dependent. In the rest of the PSM these treated explants also showed either no expression of *Hes7* at all or only a restricted weak caudal domain in the tail bud (n = 14, Figure 7D), which is reminiscent of the *Hes7* pattern of expression observed in the *Psen1*−/−;*Psen2*−/− embryos (Figure 6E). As mentioned above, *Hes7* expression in the caudal PSM is initiated by FGF activity [34],[35]. Therefore, if Notch and FGF activities are blocked simultaneously *Hes7* expression should disappear completely from the PSM. Consistent with this idea, when half embryo samples were cultured in the presence of both DAPT and SU5402, a drug that blocks FGF signalling, the expression of *Hes7* was completely abolished in the caudal PSM (n = 10, Figure 8G). Drug treatment with DAPT/LY411575 also drastically reduced *Axin2* expression throughout the PSM as compared to untreated controls, and when detected it was restricted to a weak caudal domain (n = 14, Figure 7G). These data indicate that drug treatment blocked the rostral progression of cyclic expression of both the Notch-based gene *Hes7*, as well as that of the Wnt-regulated gene *Axin2* across the PSM. To test the effect of DAPT/LY411575 treatment on somite formation we cultured E8.0–8.5 wild type mouse embryos for 18–20 hours using an *in vitro* roller culture system in the presence or absence of the drug. During this period of time control embryos formed 9–10 extra somites (n = 46, Figure 7B, 7E, 7H, 7K, 7N, and 7Q), however embryos treated with Notch-blocking drugs formed a very limited number of somites, varying from 0 to 2 (n = 65, Figure 7C, 7F, 7I, 7L, 7O, and 7R). As expected, drug-treated embryos did not show *Lfng* expression (Figure 6C). In addition, none of the treated embryos expressed *Hes7* in the rostral or medial PSM and in those that did show expression it was very weak and restricted to the caudal end of the tail bud (n = 9, Figure 7F). Likewise, none of these treated embryos expressed *Axin2* in the rostral PSM but a proportion showed some weak expression in the caudal domain (n = 8, Figure 7I). To test if FGF-dependent cyclic genes are also affected under these conditions we evaluated the expression of *Snail1*, *Dusp6* and *Sprouty2* and found that their expression seems to be not affected following a 3 hour exposure to Notch-blocking drugs (n = 10, n = 8 and n = 9 respectively, Figure 7J, 7M, and 7P), but they were severely downregulated in the PSM after overnight incubation with the drugs (n = 8, n = 8 and n = 11 respectively, Figure 7L, 7O, and 7R). Thus, these results are consistent with those obtained by analysis of the *Psen1*/*Psen2* double mutant embryos and show that, at least in the mouse, Notch activity is critical for both the maintenance and rostral progression of oscillations of the segmentation clock along the PSM and for somite formation.

Because of the oscillatory nature of cyclic gene expression in the caudal PSM of normal non-treated embryos it is possible that the remaining expression of these genes observed in the tail bud region after treatment with the drugs is indicative of the existence of an FGF/Wnt based pacemaker operational and dynamic in the absence of Notch activity. Under this paradigm the oscillations might be generated in the primitive streak/tail bud by a Notch-independent mechanism and Notch would then act to propagate these oscillations along the PSM. To test the possible existence of this Notch-independent pacemaker we used the half embryo assay and treated both halves with DAPT or LY411575 for 3 hours, then fixed one half and cultured the second half for 1 additional hour before fixation. When we performed this analysis we found that the expression of *Lfng* was completely absent in both halves, as expected (n = 3, Figure 8A). Under these conditions, if the expression of cyclic genes, such as *Hes7*, continues to oscillate in the caudal PSM after drug treatment we should find pairs of samples in which the expression domain is different in the two sides such that the cyclic gene is present in one half and absent in the other. When we analysed *Hes7* we found that the expression was weak in the two halves as compared with the intensity observed in control non-treated samples, as described above. By extending the colour revelation step we observed that *Hes7* expression was present and restricted to the caudal region of the PSM of drug-treated samples (n = 8, Figure 8B). Similarly, *Axin2* displayed a weak expression profile restricted to the caudal end of the PSM (n = 9, Figure 8C). In addition, we observed that *Snail1*, *Dusp6* and *Sprouty2* displayed similar patterns of expression in both halves (n = 12, n = 11 and n = 14 respectively, Figure 8D–8F), indicating that their expression was also not dynamic. In summary, all these results taken together demonstrate that, in the mouse embryo, in the absence of Notch signalling the expression of cyclic genes is lost in the rostral and medial PSM and is dramatically reduced in the caudal PSM where it loses its dynamism, and this is not consistent or compatible with the existence of a Notch-independent pacemaker.

## Discussion

In this study we have investigated the implication of Notch signalling in the mechanism of the mouse somitogenesis oscillator. We found that in the absence of *Lfng* the mouse embryo is still able to display dynamic expression of all cyclic genes analysed. In the absence of *Hes7*, however, only the expression of FGF and Wnt-regulated cyclic genes is still dynamic in a PSM that displays non-cyclic Notch activity along its extension. Surprisingly, in the absence of RBPj there is still some RBPj-independent Notch activity, as evidenced by the expression profile of *Hes7*. Our data show that the double mutant embryos *Psen1*−/−;*Psen2*−/− develop in the complete absence of Notch activity and they do not form somites or display oscillatory gene expression, as evidenced by the lack of expression of cyclic genes along the PSM. Similar defects are produced in wild type embryos cultured in the presence of Notch-blocking drugs. We propose that, contrary to what happens during zebrafish development, in the mouse embryo Notch activity, cyclic or non-cyclic, is critically required both for the maintenance of the somitogenesis oscillator and for the formation of the somites (Figure 8H).

It has been previously shown that Lfng and Hes7 are two important components of the Notch pathway and interfering with their functions seems to affect the somitogenesis oscillator [19],[20],[24], an idea supported by the phenotype displayed by *Lfng*−/− and *Hes7*−/− embryos [17]–[19]. Homozygous mutant embryos have clear somitic abnormalities that later in development result in skeletal malformations of vertebrae and ribs. However, the fact that these mutant embryos make somites at all indicates that the somitogenesis oscillator may still be producing oscillations and generating periodicity in the absence of these two proteins. A first analysis suggested that the Notch pathway was upregulated along the entire PSM of *Lfng*−/− embryos, as judged by the expression of Notch-dependent cyclic genes, although a more careful analysis revealed dynamic *Hes7* expression. The existence of this dynamism in the PSM of the *Lfng*−/− embryos is corroborated by the results obtained by immunostaining with the anti-Hes7 and anti-NICD antibodies that demonstrated there is periodic production of *Hes7* mRNAs and periodic production of NICD. Based on the patterns of expression described in the literature the induction of all Notch target genes in the PSM is largely generated by the interaction of Notch and the Delta family of ligands [18], [31], [56]–[59]. Our data indicates that in the mouse PSM Delta-driven Notch signalling can occur in the complete absence of Fringe activity with an intensity equivalent to when Fringe is present. We cannot exclude, however, the formal possibility that in the PSM of the *Lfng*−/− embryos another enzyme can substitute for Lfng in order to maintain the activity of the Notch pathway. Further analysis will be required to clarify the situation.

In contrast to *Lfng*−/− embryos, *Hes7*−/− embryos do not show cyclic expression of the Notch downstream target genes *Lfng* and *Nrarp*, and NICD is expressed in a rostro-caudal gradient, which is not consistent with Notch signalling being dynamic. On the other hand, the fact that the expression of FGF/Wnt-based cyclic genes is still dynamic in the *Hes7*−/− PSM indicates the FGF/Wnt-components of the somitogenesis oscillator are still operating in these embryos, which is likely to underlie the generation of periodicity and the formation of irregular somites in these embryos. A similar explanation may underlie the existence of somites in a transgenic mouse line that expresses activated non-cyclic Notch throughout the PSM (T-NICD) and shows no dynamic expression of Notch genes but, nevertheless, the cyclic expression of *Axin2* is unaffected [13]. Thus, in both *Hes7*−/− embryos and in the T-NICD transgenic embryos [13] Wnt-based genes continue to oscillate in a background of non-cyclic Notch activity. It remains unclear whether the presence of somites and the existence of cyclic gene expression in these two genetic backgrounds, *Hes7*−/− and T-NICD, are due to the combination of non-cyclic Notch together with cyclic Wnt signalling or whether cyclic Wnt alone is sufficient to account for this. The analysis of *Rbpj*−/− embryos, thought to develop in the absence of Notch activity, failed to resolve this issue since, surprisingly, the results show that at least one Notch-related cyclic gene, *Hes7*, is still expressed with different patterns of expression along the PSM suggesting its expression is still dynamic. While it remains a formal possibility that this residual *Hes7* expression is Notch-independent the fact that we report NICD is present at low levels in the PSM of the *Rbpj*−/− embryos strongly support that the *Hes7* expression is a consequence of a poorly defined RBPj-independent Notch activity in this tissue [21].

When we analysed the phenotype of the *Psen1*−/−;*Psen2*−/− embryos we found that they do not display any kind of activity of the segmentation clock, as suggested by the lack of different patterns of expression of different Notch-based, Wnt-based and FGF-based cyclic genes, and because they do not form somites. We also found that NICD is not produced in these embryos, a clear indication that these double mutant embryos really develop in the absence of Notch signalling. We can not rule out the formal possibility that there is a Notch-independent γ-secretase activity implicated in segmentation [27]. However, we think this is unlikely because amongst the list of type I transmembrane proteins known to be substrates for γ-secretase [60],[61] only the Notch components have been described to be implicated in the oscillator involved in somitogenesis. In addition, when we treated samples from wild type mouse embryos *in vitro* with drugs that block Notch cleavage we also inhibited the dynamic expression of all cyclic genes and the generation of somites beyond those already determined or in the process of being formed in the rostral PSM at the time of treatment (1 or 2). Thus, the generation of temporal and spatial periodicity in the mouse embryo absolutely requires Notch activity. In the absence of all Notch activity no oscillations occur and no somites are formed.

Feller *et al.* recently reported that NICD is not detected in the PSM of *Pofut1*−/− embryos, a mutant mouse line carrying a mutation in another relevant component of the Notch pathway, although these embryos are nevertheless still able to generate a significant number of irregular somites [13]. The authors concluded that these *Pofut1*−/− mutant embryos develop and generate somites in the absence of Notch signalling. One explanation for the difference in their interpretation compared to ours is the detection limit of our respective assays for NICD. Indeed the expression of NICD we detect in *Rbpj*−/− embryos, which also develop a limited number of somites, is very weak and could easily be overlooked. As yet an analysis of the expression profile of different Notch-based cyclic genes, including *Lfng* and/or *Hes7*, has not been performed with the *Pofut1*−/− embryos. Further investigation will be required to clarify these discrepancies.

The only *Hes7* expression domain remaining in the *Psen1*−/−; *Psen2*−/− embryos or in wild type embryos after treatment with Notch-blocking drugs is that located at the caudal end of the PSM and this has shown to be dependent on FGF signalling [34],[35]. In principle, the expression of *Hes7* in this region could be consistent with the existence of a Notch-independent pacemaker implicated in the initiation of the oscillations. However, the results produced after culturing half embryo explants from the same embryo for different periods of time in the presence of Notch-blocking drugs show that absence of Notch activity leads to a loss of dynamism in the expression of *Hes7* and *Axin2* in the caudal PSM, which indicates that at least in the mouse embryo there does not appear to be a Notch-independent pacemaker. It will be of great interest to study the mechanism by which Notch activity modifies the regulation of non-cyclic *Hes7* and *Axin2* in the caudal PSM such that it becomes oscillatory.

As mentioned above, it is widely accepted that in the zebrafish embryo the main function of Notch is to synchronise the oscillations of *her* cyclic genes and that Notch inhibition does not interrupt the generation of oscillations and the resulting segmented body plan [7]–[11],[62]. The present study does not provide evidence for a role for Notch in synchronizing the mouse somitogenesis oscillator, but it also does not seem to preclude such a role. Nevertheless our data clearly indicate that in mouse this signalling pathway plays a critical central role in the mechanism of the segmentation clock, which is not the case in zebrafish. This crucial difference in the role for Notch during mouse and zebrafish somitogenesis could be due to species-differences in the complexity of the core mechanism of the segmentation clock; an idea supported by the fact that in mouse the oscillator mechanism drives periodic expression of cyclic genes from three signalling pathways whereas in zebrafish the mechanism seems solely based on the oscillations of *her* genes which are both Notch and FGF dependent [2],[36]. So far no Wnt-based cyclic genes have been reported in the zebrafish.

In summary, our data show that in the mouse embryo Notch signalling is absolutely required to generate periodicity by the somitogenesis oscillator, as evidenced by the expression of cyclic genes and the formation of somites. The different signalling pathways implicated in this oscillator mechanism all appear to be interconnected via Notch signalling. A better knowledge of these reciprocal interactions will be of great relevance to gain a deeper understanding of the fundamental workings of this oscillatory mechanism.

## Material and Methods

### Explant culture and mouse embryo culture

Wild type CD1 *Mus musculus* embryos were obtained from timed mated pregnant females between 8.0 and 10.5 days postcoitum (dpc). *Lfng−/−*, *Hes7−/−*, *Rbpj−/−* and *Psen1−/−;Psen2−/−* embryos were obtained and genotyped by PCR analysis of the yolk sacs as described [18],[19],[39],[41]. For half embryos analysis E9.5–10.5 mouse embryos were isolated and the caudal portion was divided into two halves by bisecting the tissue along the midline. Explants were cultured on medium composed of DMEM/F12 supplemented with 10% fetal bovine serum, 10 ng/ml bFGF, and 50 U/ml penicillin/streptomycin. At the end of the culture period the explants were transferred into 4% paraformaldehyde fixative solution and then analysed by *in situ* hybridisation for gene expression. Four different series of experiments were performed: (A) One half explant was fixed and the other half was cultured for 60 minutes. (B) The two halves were cultured for 3 hours in medium in the absence or presence of either 100 µM DAPT (Calbiochem) or 100 nM LY411575 to inhibit the Notch activity, or the equivalent volume of DMSO as control. (C) The two halves were cultured for 3 hours in medium in the presence or absence of a mix of 100 µM DAPT plus 50 µM SU5402 (Calbiochem). (D) The two halves were cultured for 3 hours in medium in the presence of 100 µM DAPT or 100 nM LY411575, then one half was fixed and the second half was cultured for 1 additional hour before fixation. Whole embryo mouse culture was performed as previously described [63],[64] using wild type embryos. In short, E8.0–8.5 mouse embryos with their membranes intact were cultured for about 18–20 hours in standard whole embryo roller culture conditions: 50% rat serum in F12 medium plus 1 mM sodium pyruvate, 2 mM glutamine and non-essential amino acids at 37°C with 5% CO_2_. Media was supplemented with either 100 µM DAPT or 100 nM LY411575 or the appropriate amount of DMSO as control. All animals were handled in strict accordance with good animal practice as defined by the relevant national and/or local animal welfare bodies, and all animal work were approved by the ethical committees for experiments with animals of the University of Dundee (UK), Nara Institute of Science and Technology (Japan), University of Leuven (Belgium) and Centro Nacional de Investigaciones Cadiovasculares (Spain).

### Whole-mount *in situ* hybridisation

Mouse intronic and exonic *Lfng*, intronic and exonic *Hes7*, *Axin2*, *Mesp2*, *Snail1*, *Uncx4.1*, *Dusp6* and *Sprouty2* probes were prepared as described [16],[20],[23],[30],[32],[37],[65],[66]. Whole-mount *in situ* hybridisation was done basically as described [67]. The following modifications to this protocol were used for intronic probe *in situ* hybridisation. Samples were hybridised with probe for 40 hours in a low stringency hybridisation mix (50% formamide, 5× SSC, 5 mM EDTA, 50 µg/ml tRNA, 0.2% Tween-20, 0.1% SDS, 100 µg/ml heparin) and post-hybridisation washes were performed in post-hybridisation buffer (50% formamide, 0.1% Tween-20, 1× SSC). Samples were processed either by hand or using the InsituPro VS Robot (Intavis AG). All images were captured on a Leica MZ16 APO microscope using a Jenoptik camera. Images were recorded using Openlab software version 4.0.3.

### Quantitative real-time RT–PCR

The reaction was accomplished in the presence of SYBR Green Supermix (BioRad) and the reactions were measured in a Mastercycler ep realplex (Eppendorf) using the following cycling conditions: 95°C for 5 min, 40 cycles at 95°C for 15 sec and 53°C for 60 sec. Primers to quantify *Hes7* mRNA levels were designed using Primer3. The two primers used were 5′-GAAGCCGTTGGTGGAGAAG-3′ and 5′-GGCTTCGCTCCCTCAAGTAG-3′. Normalization was performed against β-actin amplified using the primers 5′-GGCTGTATTCCCCTCCATCG-3′ and 5′-CCAGTTGGTAACAATGCCATGT-3′.

### Alcian Blue and Alizarin Red staining

E18.5 mouse embryos were fixed in 95% ethanol overnight at room temperature and stained with 150 mg/ml Alcian Blue in 1∶4 mixture of acetic acid and 95% ethanol at RT for 24–48 h. After washing with 95% ethanol for 1 h, the embryos were treated with 1% KOH for 24 h with several changes. Embryos were subsequently stained with 75 mg/ml Alizarin Red S in 1% KOH solution for 12–24 h and cleared in a solution of 20% glycerol and 1% KOH for a week with daily changes. Samples were transferred to 50% glycerol, 50% ethanol for photography and storage.

### Immunohistochemistry

Whole-mount immunohistochemistry with anti-Hes7 and anti-NICD antibodies was performed as described previously [20],[27],[46]. Briefly, for analysis with the anti-Hes7, embryos were fixed with 4% paraformaldehyde in PBS at 4°C for 3 h and treated with 0.1% H_2_O_2_ overnight. Then the embryos were incubated with anti-Hes7 antibody (1∶100 diluted) at 4°C for 3–5 days and next with HRP-conjugated anti-guinea pig IgG (Chemicon) overnight at 4°C. The peroxidase deposits were visualized by 4-chloro-1-naphthol. For analysis with the anti-NICD on transversal sections 8-µm paraffin-embedded sections were immersed in 10 mM sodium citrate pH 6.0 and boiled 10 min to enable antigen retrieval. Immunostaining was performed with cleaved Notch1 antibody (Val1744, 1∶100 diluted, Cell Signaling Technology) overnight at 4°C, followed by biotinylated anti-rabbit IgG antibody (1∶100 diluted, Vector Laboratories) for 60 min at RT. Finally, the signal was amplified in two steps; first with avidin/biotin-HRP (ABC kit, Vector Labs) for 60 min at RT and then with Tyramide-Cyn3 (NEL 744, Perkin Elmer).

### Western blot analysis

Samples were prepared with caudal fragments of E9.5–10.5 mouse embryo control or treated with drugs and with mouse embryonic fibroblast derived from *Psen1*−/−;*Psen2*−/− embryos [47]. 20 µg of protein samples were used to perform electrophoresis in MOPS running buffer. Gels were then blotted and the resulting membrane was incubated with the anti-NICD antibody (Val1744, 1∶1000 dilution, Cell Signaling Technology) overnight at 4°C followed by anti-rabbit-HRP antibody (1∶1000) for 60 min at RT and then standard ECL revelation (Pierce). α-Tubulin staining was preformed with a 1∶20000 dilution (Abcam, ab7291) followed by a 1∶2000 dilution of anti-mouse-HRP antibody.
